# Nanoparticles for Lymph Node-Directed Delivery

**DOI:** 10.3390/pharmaceutics15020565

**Published:** 2023-02-08

**Authors:** Jaeseong Lee, Sungtaek Kang, Hyeseon Park, Jeong Gil Sun, Eun Chae Kim, Gayong Shim

**Affiliations:** School of Systems Biomedical Science and Integrative Institute of Basic Sciences, Soongsil University, Seoul 06978, Republic of Korea

**Keywords:** lymphatic delivery, nanoparticles, cancer therapy, cancer metastasis

## Abstract

Lymph nodes are organs that control immune cells and provide a major pathway for primary tumors to metastasize. A nanoparticles-based strategy has several advantages that make it suitable for achieving effective lymphatic delivery. First, the size of nanoparticles can be tailored to meet a size range appropriate for lymphatic migration. In addition, functionalized nanoparticles can target cells of interest for delivery of drugs or imaging probes. Existing lymph node contrast agents map all lymph nodes regardless of metastasis status; however, by using nanoparticles, it is possible to selectively target lymphatic metastases. Moreover, using functionalized nanoparticles, it is possible to specifically deliver anticancer drugs to metastatic lymph nodes. In this review, we introduce the use of nanoparticles for lymphatic mapping, in particular highlighting design considerations for detecting metastatic lymph nodes. Furthermore, we assess trends in lymph node-targeting nanoparticles in clinical practice and suggest future directions for lymph node-targeting nanoparticles.

## 1. Introduction

Lymph nodes are nodules located between lymphatic vessels and are thus part of the lymphatic system that forms a network distributed throughout the body [1]. Lymph nodes manage the generation, proliferation, and activation of lymphocytes and are the organs where adaptive immune responses take place [2]. Lymph nodes are also major routes for metastasis of primary tumors; in fact, more than 90% of cancer cells migrate through a lymph node [3]. Malignant tumors show differences in prognosis depending on lymph node metastasis. Therefore, lymph node metastasis is an important factor in determining the prognosis of cancer patients. Dissection of the lymph nodes around a tumor is considered standard treatment, and metastatic lymph nodes play an important role in tumor, node, metastasis (TNM) staging decisions [4]. After locating and excising the sentinel lymph node (SLN), the site where tumor metastasis first occurs, cancer metastasis is determined through a biopsy. Notably, detecting SLNs is important from the standpoint of chemotherapy.

Through SLN mapping, the lymph nodes around the tumor are accurately identified and removed, followed by a histological examination to confirm the presence of lymph node metastasis and make it possible to diagnose metastatic cancer. SLN imaging methods include nuclear medicine molecular imaging, optical imaging, and near-infrared fluorescence imaging [5]. Evans blue or methylene blue for optical imaging is relatively invasive because imaging is difficult and lymph nodes must be exposed during surgery to check the staining of the lymph nodes. The method using contrast agents containing radioactive isotopes has good imaging sensitivity, but it is difficult to escape from productivity and handling problems. In particular, safety issues such as side effects caused by irradiation should be considered. Among fluorescent dyes used for imaging, the FDA-approved indocyanine green (ICG), which absorbs light in the near-infrared region, is the most suitable for SLN mapping [6]. ICG has a low possibility of inducing autofluorescence and allows for relatively high-penetration near-infrared imaging [7]. Compared to radioactive isotopes, it has no side effects and is competitively priced. However, it does have limitations of low photostability, short half-life, and non-specific lymph node staining.

**Figure 1:**
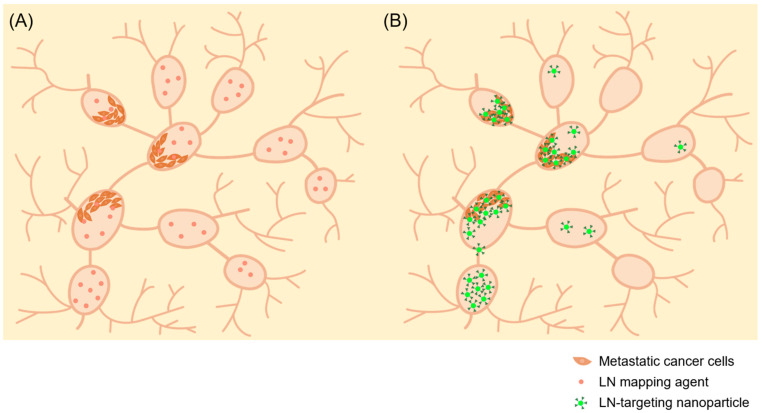
Nanoparticles targeting metastatic lymph nodes. (**A**) LM mapping agent has no targeting ability on metastatic lymph nodes. (**B**) Functional nanoparticles specifically detect metastatic lymph nodes, enabling minimal lymphadenectomy and metastatic cancer-specific therapeutic drug delivery.

Currently used nanoparticles for lymphadenography can detect lymph nodes, but cannot distinguish metastatic lymph nodes [5,8,9]. Lymph node dissection as a strategy for preventing cancer metastasis can cause side effects such as lymphedema and a reduction in the patient’s quality of life [10]. Detecting metastatic lymph nodes and minimally resecting them can reduce the risk of treatment complications. In addition, developing nanoparticles capable of detecting metastatic lymph nodes could achieve targeted treatment through metastatic cancer-specific delivery of anticancer drugs (Figure 1).

Cell-mediated drug delivery has been actively used in research on lymph node-targeting nanoparticles, including vaccines and metastatic cancer treatment. Locally injected nanomaterials can be taken up by dendritic cells and transported to lymph nodes, regardless of size. Microneedle formulations for sustained release have also been extensively studied for vaccine development. In this review, we address nanoparticles with the ability to migrate directly to the lymph node rather than cell-mediated lymph node targeting.

## 2. Principles of Lymphatic Delivery

**Figure 2:**
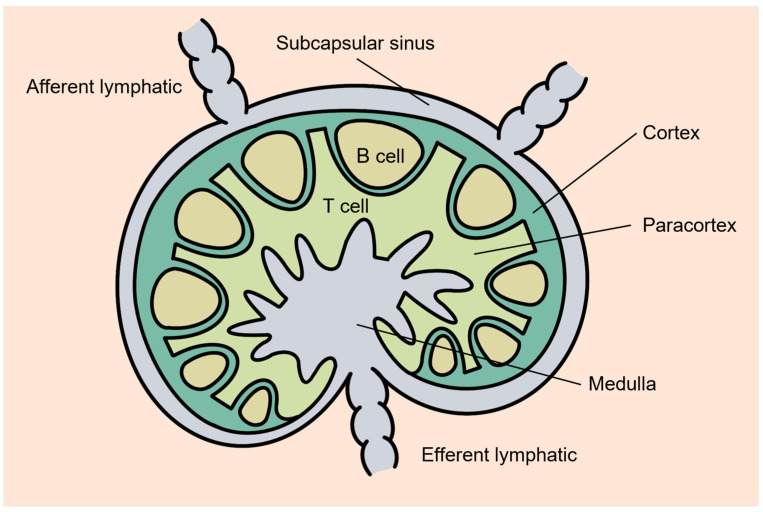
Structure of a lymph node. Lymph nodes, most of which are kidney-shaped sacs, are located between lymphatic vessels. The outer part of the lymph node is called the cortex and contains B cells that produce antibodies. The remainder of the cortex contains T cells that circulate throughout the body and monitor immune responses. Lymph flows in through the afferent lymphatic duct, which is connected to the convex side of the lymph node, and drains through the efferent lymph vessel.

Particles injected into the body interact with various types of immune cells present in the lymph nodes to induce an immune response [11]. The lymph node acts as a filter for body fluids, and particles that migrate from other tissues enter the lymph node through the afferent lymphatic duct (Figure 2). The lymphatic vessel-accessibility of nanoparticles introduced into the blood differs depending on the nanoparticle size. Particle size between 10 and 100 nm in diameter can target the lymphatic drainage due to the fact of rapid clearance of small molecules from the blood into the lymphatic system [1,11,12]. Particles with larger diameters remain in the interstitial matrix and only access the lymphatic fluid with difficulty. Within the size range that can migrate to lymphatic vessels (10–100 nm), relatively smaller nanoparticles accumulate in more metastatic lymph nodes. Particle properties other than size have also been reported to affect lymphatic delivery [12]. Flexible nanoparticles are more advantageous than rigid nanoparticles in penetrating into the interstitial space and entering lymphatic vessels. In addition, the interstitial matrix is an environment in which particles with negative surface charges can easily migrate.

## 3. Nanovehicles for Lymphatic Delivery

The development of lymph node-targeting nanoparticles relies on research designed to select nanoparticle materials suitable for migration to lymph nodes or modify materials to incorporate lymph node-specific ligands as indicated in Table 1.

### 3.1. Environment-Responsive Materials

Imaging nanomaterials containing pH-responsive polymers that are switched on in metastatic SLNs have been studied [13,41]. These nanomaterials were designed to achieve pH-sensitive imaging by connecting luminol and a fluorescent probe to an ionizable block copolymer that forms self-assembling micelles. These nanoparticles have an average diameter of 35.2 ± 2.6 nm at neutral pH in vivo and exist in a switched-off state due to aggregation-induced quenching. However, when introduced into inflammatory macrophages of the metastatic SLN, the nanoparticles disassemble in the acidic environment of phagosomes and recover their luminescence.

Another study has also reported detection of metastatic lymph nodes using nanoparticles that respond sensitively to pH [14]. The methacrylate micellar nanoparticles used in this study are capable of structural interconversion between the micelle form and the cationic monomer form according to the proton concentration in the environment. At pH 7.4—the pH in benign lymph nodes—the indocyanine green that collects inside the micelle is switched off by Förster resonance energy transfer (homo-FRET)-mediated quenching. In contrast, the fluorescence signal of indocyanine green is amplified in metastatic lymph nodes, reflecting operation of the principle that micelles disassemble in response to cancer acidosis.

Hyaluronic acid, a key component of the extracellular matrix, was degraded to increase the delivery rate of nanoparticles to metastatic lymph nodes [18]. In the tumor microenvironment where hyaluronidase is administered, hypoxia is alleviated and the enhanced permeability and retention effect of the tumor is increased. Accordingly, the uptake of nanomicelles loaded with chlorin e6 was improved, and the combined treatment of hyaluronidase and nanomicelles effectively inhibited the growth of primary tumors. In addition, in the breast cancer metastasis model, it was confirmed that nanomicelles were effectively accumulated in the metastatic lymph node of the hyaluronidase-treated area. This result shows that the nanoparticle delivery rate is determined by the enhanced permeability and retention effect even in metastatic lymph nodes.

pH-responsive polymers [13], which are modalities that depend on the acidic condition associated with inflammation, represent an indirect method for detecting metastatic SLNs. Combining this approach with other probes, such as a tumor-targeting ligand, would confer complementary benefits. Systems that are sensitive to cancer acidosis [14] can be turned on in response to lymph node-resident macrophage-mediated phagocytosis of nanoparticles or nonspecific endocytosis, either of which can induce a background signal.

### 3.2. Tailored Nanoparticles

Mannan-functionalized nanoparticles have been reported to target mannose receptors expressed in antigen-presenting cells [33,35]. Mannan is a polymer composed of mannose and is a component of the cell wall of microorganisms. Park and colleagues performed MR imaging of lymph nodes using mannan-coated superparamagnetic iron oxide nanoparticles [35]. Metastatic lymph nodes can be identified by MR imaging using the fact that mannan-coated metal nanoparticles are preferentially absorbed by antigen presenting cells, active macrophages and dendritic cells. The same group prepared gold nanoparticles using mannan as a stabilizer and compared them to existing citrate-capped gold nanoparticles [33]. Mice topically administered with mannan-capped gold nanoparticles significantly increased X-ray contrast for imaging of popliteal lymph nodes. Targeting of mannan-functionalized nanoparticles was observed by mannose receptor-mediated endocytosis.

Copper sulfide (CuS) nanoparticles targeting gastric cancer cells overexpressing the integrin, αVβ3, have been used for metastatic lymph node imaging and image-guided photothermal therapy [32]. Because CuS nanoparticles absorb X-rays, their biodistribution can be detected by computed tomography (CT) scanning. A near-infrared fluorescence (NIRF)/CT dual-imaging modality was also designed by modifying the nanoparticle surface to contain Cy5.5. In this application, cyclic RGD (cRGD), a targeting ligand of αVβ3, was introduced onto the surface of CuS nanoparticles through amide bonding. Nanoparticles administered by intratumoral injection migrated to the draining lymph node and effectively inhibited lymph node metastasis through NIRF/CT image-guided photothermal therapy.

Cyclic RGD was also modified into lipid-based nanoparticles and used for photoacoustic imaging of metastatic lymph nodes [28]. N4 oligomer, an isomorphic organic semiconductor, was used as a contrast material for photoacoustic imaging, and nanoparticles were formed by precipitating it with PEG lipid. To target integrin αvβ3, cyclic RGD was modified on the surface using maleimide at the PEG end. N4 oligomer nanoparticles clearly imaged the SLN with a maximum signal 10 min after intradermal injection. SLN photoacoustic imaging of the nanoparticles demonstrated photostability, retaining 45% of the maximum signal even after 60 min after in vivo injection. The nanoparticles developed in the study also acted as photothermal agents and showed higher photothermal therapeutic efficacy in triple negative human breast adenocarcinoma MDA-MB-231cells than in fibroblast NIH-3T3 cells.

An approach using a dual-targeting strategy was investigated for detecting metastatic SLNs in breast cancer [24]. This study used CD44 and scavenger receptor class B type 1 (SR-B1), which are overexpressed on tumors, for targeting. CD44 was targeted with a hyaluronic acid-conjugated lipid [42] and SR-B1, which is expressed in breast cancer and is known to bind to high-density lipoprotein (HDL), was targeted using an HDL-mimicking peptide [43]. A 5 kDa form of hyaluronic acid showed superior lymph node migration ability compared a 15 kDa form. Dual-targeting nanoparticles showed a distinct distribution to tumor metastatic SLNs relative to normal or inflamed lymph nodes.

The transport efficiency of lymphatic endothelial cells was observed by adjusting the PEG density on the nanoparticle surface [15]. The transport rate of PEGylated nanoparticles was more than 50 times higher than polystyrene nanoparticles that were not modified with PEG. The higher the density of PEG, the higher the transport rate of nanoparticles. Particles of two sizes (100 or 40 nm) were compared in the size range of nanoparticles capable of passing through lymphatic vessels, and no difference in transport rate was observed according to size. In addition, pegylated nanoparticles had a significantly higher lymph node accumulation rate compared to plain nanoparticles.

Nanoparticles entering lymph nodes through affrent lymphatic vessels can stay in the subcapsular sinus, but penetration to the cortex and paracortex is difficult. Nanoparticles for lymph node delivery using a thiol-responsive moiety have been studied [16]. Poly(propylene sulfide) nanoparticles were loaded with CpG adjuvant using oxanorbornadiene or disulfide linker. Nanoparticles reaching the subcapsular sinus have different fates depending on the drug-loaded linker. Nanoparticles with drugs linked by disulfide bonds cannot pass through the lymph nodes but can be absorbed into the lymph nodes through phagocytosis by macrophages. In contrast, a drug linked to a thiol-reactive oxanorbornadiene linker is capable of penetrating the ducts into the cortex and volume of the lymph node after release.

There has been a study targeting circulating tumor cells, the source of distant metastasis, using the neutrophil membrane [17]. Inflammatory neutrophils can target circulating tumor cells, but their half-life is short, making them difficult to use as cell therapy. In this study, the plasma membrane of neutrophils was isolated and coated on poly(lactic-co-glycolic acid) nanoparticles loaded with carfilzomib. Among the proteins present in the membrane of neutrophils, L-selectin, Lymphocyte function-associated antigen 1 (LFA-1), and β1 integrin were involved in tumor targeting by interacting with CD44, intercellular adhesion molecule 1 (ICAM-1), vascular cell adhesion molecule 1 (VCAM-1) on the surface of circulating tumor cells, respectively. Intravenously administered neutrophil-mimicking nanoparticles not only prevented the formation of early metastases through targeted delivery of the proteasome inhibitor carfilzomib, but also inhibited the progression of cancers that had already metastasized to the lungs.

An antibody was modified on the surface of up-conversion nanoparticles to target metastatic gastric cancer lymph nodes [38]. NaGdF4:Yb,Er nanoparticles were coated with maleimide-PEG and the surface was modified with MGb2 antibody by click chemistry. PEG coating increases retention in the blood and is helpful for lymphatic migration. MGb2 antibody recognizes the trafficking protein, kinesin-binding 1 (TRAK1), which is expressed in more than 80% of gastric carcinomas. Antibody-decorated nanoparticles showed a stable size distribution in the buffer for 1 year. MGb2 Ab-decorated up-conversion nanoparticles had specific binding to gastric cancer SGC7901 cells compared to IgG control-decorated nanoparticles. In addition, this nanoparticle proved that metastatic lymph node imaging is possible in an orthotopic model constructed with SGC7901-luc.

Because the RGD ligand targets αVβ3, which is also expressed in normal cells, including macrophages, it is possible for CuS to exhibit an undesirable distribution [32]. Therefore, the level of αVβ3 expression in the target cancer will be an important application criterion. This dual-targeting strategy [24] is suitable for specifically targeting the tumor metastatic SLN, but more in-depth research is needed to determine whether physical hindrance between the two ligands changes their respective targeting ability. Dir-BOA [24], which is capable of both fluorescence imaging and photoacoustic imaging, has also been used. Accordingly, it is important to select a probe with actual development potential for its intended clinical use.

## 4. Anti-Metastatic Efficacy of Lymph Node Directed Delivery of Nanoparticles

### 4.1. In Vivo Antimetastatic Efficacy in Experimental Animal Models

Polymer-based nanoparticles targeting metastatic lymph nodes of gastric cancer have been studied [19]. In this study, ICG-loaded polymersome was formed by mixing poly(L-lactic acid) conjugated with ICG and poly(sarcosine)–poly(L-lactic acid). Polymersome was observed to be distributed in metastasized popliteal lymph nodes after intravenous administration to a metastatic lymph node animal model using human gastric carcinoma MKN45 cells. In contrast, intravenously administered free ICG showed almost no fluorescence intensity in the lymph nodes, regardless of metastasis, and the distribution of polymer nanoparticles was not observed in the lymph nodes without metastasis. Observations were made 48 h after administration, so it is difficult to observe a free ICG signal. As a result of photodynamic therapy using the photosensitivity of loaded ICG, the volume increase of the metastatic popliteal lymph node was delayed and the proportion of apoptotic cells in the lymph node increased.

The metastatic lymph node suppression effects of doxil, a nano-anticancer drug, and platinum drug-incoporated nanomicelles were compared in a melanoma metastasis model [20]. In this study, nanomicelles with an average diameter of 30 nm suitable for lymphatic migration and 70 nm nanomicelles with a size similar to doxil were prepared. Platinum (II) analog (dichloro(1,2-diaminocyclohexane)platinum(II)), which is effective for inhibiting metastatic lymph nodes, was loaded inside nanomicelles. Then, 30 nm nanomicelles were systemically administered to two melanoma models in which primary tumors were maintained or surgically removed. As a result, 30 nm nanomicelles were distributed more than four times more in metastatic lymph nodes than in normal lymph nodes, regardless of the presence or absence of primary tumors. Microdistribution imaging of metastatic lymph nodes showed that 30 nm nanomicelles penetrated more into the tumor interior than doxil. The metastatic lymph node inhibitory effect of 30 nm nanomicelles was superior to that of 70 nm nanomicelles. This finding suggests that differences in diameter can affect the rate of drug delivery to metastatic lymph nodes and the recurrence rate.

A recent study reported a strategy for targeting both metastatic lymph nodes and primary tumors through in situ conversion of particle size [25]. In this study, paclitaxel-encapsulated lipid micelles with an initial diameter of about 25 nm, an appropriate size for lymphatic migration, were prepared and their surfaces were functionalized with azide/alkyne, allowing micelles to be linked to each other through a click chemistry reaction. Following administration in mice, micelles that reached the tumor were converted into larger micelles with a diameter of about 100 nm through click cycloaddition, mediated by intratumorally injected catalyst. Because of this transformation, nanoparticles showed sustained retention in the tumor and exhibited increased tumor-reduction efficacy compared with the catalyst-injected group (25 nm micelle), reducing lymph node metastasis by 66.7%.

Liposomes encapsulated with antigens and immune enhancers were orally delivered to lymph nodes through gastrointestinal absorption [26]. Ovalbumin and poly I:C, a model antigen and a nucleic acid-based adjuvant, were loaded into cationic liposomes. The surface of liposomes was coated with two sulfated glycosaminoglycan derivatives. First, glycocholic acid (a kind of bile acid) conjugated chondroitin sulfate was used to increase the bioavailability after oral administration. Second, the nanoparticles were coated with mannose-linked chondroitin sulfate to induce dendritic cell-specific cellular uptake in lymph nodes. Liposomes coated with mannose and glycocholic acid migrated intact to the lymph nodes. Oral administration of these nanoparticles further increased dendritic cell maturation compared to intradermal administration. Moreover, the induction of B16F10 melanoma or CT26 colorectal cancer was significantly reduced in nanoparticle-vaccinated mice. In particular, the formation of melanoma and colorectal cancer was prevented by 100% and 60%, respectively, in mice administered with the nanoparticles two weeks ahead.

Liu et al. [27] performed in situ delivery using LN accumulation of endogenous albumin. By comparing cholesterol, monoacyl lipid, and diacyl lipid derivatives, it was observed that the candidate lipid conjugated CpG adjuvant with diacyl chain binds well to albumin. In addition, tumor antigen peptides derived from SIV Gag, Trp2, or E7 were linked to PEG-diacyl lipids to compare albumin-loading abilities. Conjugates (C18) with a long spacer PEG length and a high number of carbon atoms in lipids showed a high rate of LN accumulation. Mice immunized with CpG adjuvant or antigen peptide-linked lipids induced the expansion of antigen-specific CD8+ T cells. In TC-1 tumor-bearing mice expressing E7 protein, the group administered with CpG/peptide-lipid derivatives showed a much better antitumor effect than the group administered with original CpG and peptide.

Size-shiftable micelles effectively increase tumor retention [25]. However, this approach is limited by its narrow applicable range because of potential difficulties in injecting the catalyst into the tumor because of the tumor’s location. In cases where the size conversion principle reflects operation of specific factor in the tumor microenvironment, this approach should prove more versatile. Since most nanoparticle formulations are studied for intravenous or local administration, the development of nanoparticles for oral administration is a meaningful study in terms of patient pain relief [26]. However, there is a high possibility of cargo loss, which is vulnerable to the harsh environment of the gastrointestinal tract. In addition, the nanoparticles might act on the whole body and induce the maturation of non-specific dendritic cells. To avoid immune-related side effects, an advanced targeting strategy is required. LN-specific delivery, which obtains albumin by imparting lipophilic properties to vaccines and adjuvants in soluble form, was effective in immunotherapy [27]. Evans blue [44] and ICG [45], which are dyes used for SLN mapping, have also been reported to bind to albumin after administration, and hitchhiking research using them seems to be possible. However, considering the possibility of binding to plasma proteins other than albumin, a method of first formulation with albumin in vitro can be tried.

### 4.2. Clinical Study

Nanoparticles that target lymph nodes are currently available for use as contrast agents for SLN mapping (Table 2). Lipiodol Ultra Fluid, the first iodinated contrast agent developed, is composed of a mixture of iodized fatty acids from poppyseed oil [8,46]. Nanoparticles containing radioactive isotopes or iron oxide are additionally used as contrast agents. However, in each case, these nanoparticles are formulated for the purpose of stabilizing the tracer; nanoparticles that target metastatic lymph nodes have not yet been developed.

In one attempt to detect SLNs in breast cancer patients, researchers injected patients with superparamagnetic iron oxide nanoparticles (SPION) before or after surgery [47]. SPIONs showed a detection rate of 95.6% (184 procedures). This rate is similar to that achieved using radioactive isotope 99mTc, a standard method for SLN biopsy in breast cancer patients. These researchers further found injection before surgery was more effective for SLN detection than injection after surgery.

SPIONs have also been studied for the detection of SLNs in prostate cancer patients [48,49]. These studies provided the first demonstration of the effectiveness of intraoperative SLN detection using SentiMag/Sienna+ in prostate cancer patients [48]. None of the 20 cancer patients injected with Sienna+ showed any side effects, and SentiMag detected trapped SLNs with 94% accuracy. In further studies, Winter and colleagues took advantage of the fact that Sienna+ has properties similar to those of SPIONs, which are used as negative MRI contrast agents [45]. An MRI analysis of 50 prostate cancer patients injected with SPIONs (Sienna+) revealed an SLN-detection rate of 100%. This is the same diagnostic rate achieved using the existing Sienna+ detector, SentiMag, and is significant in that this dual system allows diagnosis of SLNs without the use of radioactive isotopes.

Carbon nanoparticles have been used for lymph node mapping during cancer surgery [50,51,52]. Carbon nanoparticles injected during thyroid cancer surgery stain the thyroid gland and thyroid draining lymph nodes black. In one study, an analysis of data from 406 patients who underwent total thyroidectomy and central lymph node dissection to prevent thyroid recurrence [52] showed that the use of carbon nanoparticles as a lymph node contrast medium helped identify the patient’s parathyroid gland, reducing the incidence of temporary side effects after surgery.

Carbon nanoparticles, which are currently in use as lymph node mapping probes, are suitable materials for introducing cancer-targeting ligands [52]. Thus, the expectation is that it will be possible to develop therapeutic agents that target metastatic lymph nodes. The size of SPION particles (>80 nm) is similar to that of existing 99m technetium nanocolloids (60 nm), but SPIONs have the advantage of a more uniform diameter distribution [48]. In addition, SLNs entrapping SPIONs may show a brown discoloration, potentially providing additional identification information. However, carbon nanoparticles are not recommended for patients with iron intolerance or hypersensitivity. Micrometastases smaller than 2 mm can be detected by lymphatic mapping, but when their size becomes larger (macrometastases), the role of tumor-targeting ligands becomes important [9].

## 5. Conclusions

Nanoparticles have several advantages for lymph node drug delivery. Because of their larger size relative to typical biomolecules, they can easily migrate into lymphatic vessels. In addition, it is possible to impart lymph node-targeting ability through surface modification of nanoparticles. However, nanoparticles introduced in this manner can interact with phagocytic cells present in the sinus, but only enter the T cell zone with difficulty owing to size limitations. Therefore, the design of nanoparticles requires a consideration of the size and surface charge that can enter the lymphatic vessels, and strategies for infiltrating tumors in the lymph nodes, including tumor-specific ligands, size conversion, and lymph node environment-specific release of drugs [53,54]. Lymph nodes can be targets for vaccination as well as direct drug delivery, but this approach can induce immune tolerance, so an accurate targeting strategy is essential. There are reports that the surface of nanoparticles with negative or positive charges is advantageous for lymph node accumulation. Contrariwise, the Maisel group observed that neutral nanoparticles densely modified with PEG increase the delivery rate of nanoparticles into lymphatic vessels by controlling the cell transport mechanism [15]. Since the environment until reaching the lymph node is diverse, it is also necessary to design nanoparticles that can be switched according to the route from administration to arrival.

## Figures and Tables

**Table 1:** Nanoparticle-mediated lymphatic delivery systems. Selected studies of lymph node-targeting nanoparticles are summarized.

Category	Materials	Diameter	Surface	Cargo	Probe	Route	Anima Model	End Point	Ref.
Polymer	PC7A-luminol-pyropheophorbide-a	33.2 ± 5.9 nm (pH7.4)			Luminol/pyropheophorbide-a	S.C.	Balb/c mice bearing 4T1-GFP or CT26-GFP tumor	In vivo imaging	[13]
	Methacrylate	23–28 nm			ICG	I.V.	Orthotopic Balb/cj mice-bearing 4T1.2 tumor	In vivo imaging	[14]
	Polystyrene	100 nm or 40 nm	Polyethylene glycol (PEG)		Red fluorescent dye	I.D.	C57Bl/6J mice	In vivo imaging	[15]
	Poly(propylene sulphide)	27 ± 0.6 nm		CpG DNA	Rhodamine	I.D.	C57BL/6J mice bearing EL4-GFP tumor	In vivo efficacy	[16]
	poly(lactic-co-glycolic acid)	94.8 ± 7.6 nm	Neutrophil membrane	Carfilzomib	Coumarin-6, DiR dyes	I.V.	Balb/c nude mice bearing 4T1 tumor	In vivo efficacy	[17]
	poly (maleic anhydride-alt-1-octadecene)	about 33 nm	PEG	Chlorin e6	Chlorin e6	I.V.	Balb/c mice bearing 4T1 tumor	In vivo efficacy	[18]
	Poly (sarcosine)–poly (L-lactic acid)	40–50 nm		ICG		I.V.	Balb/c nude mice bearing MKN45 tumor	In vivo efficacy	[19]
	Poly(ethylene glycol)-b-poly(l-glutamic acid)	30 ± 2 nm or 73 ± 1 nm		Dichloro(1,2-diammino cyclohexane) platinum(II)	Alexa 647, Alexa 555 dyes	I.V.	C57BL/6J mice bearing B16F10-Luc tumor	In vivo efficacy	[20]
	Pluronic-stabilized polyphenylene sulfide	30 nm		CpG DNA, ovalbumin, Trp-2		S.C.	C57BL/6 mice bearing EG.7-OVA tumor or EL4 tumor	In vivo efficacy	[21]
	1,4-O-methacryloyl hydroquinone, coumarin comonomer	50, 100 nm		Paclitaxel	IR-813	I.D.	Yorkshire pigs	In vivo imaging	[22]
	Poly(lactic-co-glycolic acid)	80~90nm	LyP-1, PEG		Coumarin	S.C.	Balb/c nude mice bearing BxPC-3 tumor	In vivo imaging	[23]
Lipid	1,2-Dimyristoyl-sn-glycero-3-phosphocholine, 1,2-dimyristoyl-sn-glycero-3-phosphoethanolamine and 1,2-distearoyl-sn-glycero-3-phosphoethanolamine-N-[methoxy(polyethylene glycol)-2000]	~43 nm	Hyaluronic acid/HDL-mimicking peptide		DiR-BOA dye	S.C.	Albino-C57BL/6 mice-bearing 4T1-tfRF tumor	In vivo imaging	[24]
	Azide/alkyne-functionalized lipid	~25 nm		Paclitaxel	Alexa 647 dye	I.V.	Balb/c mice bearing 4T1 tumor	In vivo efficacy	[25]
	1,2-dioleoyl-3-trimethylammonium-propane and 1,2-dioleoyl-sn-glycerol-3-phosphocholine	157 ± 3 nm	Chondroitin sulfate-g-mannose and chondroitin sulfate-g-glycocholic acid	Ovalbumin/poly I:C	Fluorescence-labelled PEG-diacyl lipid	P.O.	C57BL/6 mice bearing B16F10 tumor & balb/c mice bearing Ct26 tumor	In vivo efficacy	[26]
	Endogenous albumin	-	PEG-diacyl lipid-conjugated antigen peptide	CpG DNA		S.C.	C57BL/6 mice bearing TC-1 tumor or B16F10 tumor	In vivo efficacy	[27]
	1,2-distearoyl- sn -glycero-3-phosphoethanolamine-N-[maleimide(polyethylene glycol)-2000]	≈45 nm	Cyclic arginine-glycine-aspartic acid	N4 oligomer		S.C.	Wistar rats	In vivo imaging	[28]
	Pyropheophorbide–lipid	120 ± 5 nm		Copper 64		I.V.	New Zealand white rabbits bearing VX2 tumor	In vivo imaging	[29]
	1,2-distearoyl-snglycero-3-phosphatidyl choline, 1,2-distearoyl-sn-glycero-3-phosphoethanolamine-methoxy-polyethyleneglycol	~100 nm		Perfluoropropane		I.N	MXH10/Mo/lpr mice	In vivo efficacy	[30]
	Cholesterol, 1,2-dioleoyl-sn-glycero-3-phosphocholine, dioleoylphosphatydic acid,1,2-Dioleoyl-3-trimethylammonium-propane chloride salt, 1,2-distearoryl-sn-glycero-3-phosphoethanolamine-N-[methoxy(polyethyleneglycol-2000), and 1-oleoyl-2-[12-[(7-nitro-2-1,3-benzoxadiazol-4-yl)amino] dodecanoyl]-sn-glycero-3-phosphocholine	~25 nm		Indium-111		I.V.	Balb/c mice bearing 4T1-luc2-GFP tumor	In vivo imaging	[31]
Metal	Copper sulfide nanoparticles	~21 nm	cRGD		Cy 5.5 dye	I.T.	Balc/c mice bearing MKN45 tumor	In vivo imaging	[32]
	Mannan-capped gold nanoparticles	9.18 ± 0.71 nm	Mannan			S.C.	C57BL/6 mice	In vivo imaging	[33]
	Gold nanoparticles	10, 22, 33 nm		Ovalbumin		S.C.	C57BL/6 mice bearing EG.7-OVA tumor	In vivo efficacy	[34]
	Superparamagnetic iron oxide	46.2 ± 1.9 nm	Mannan		FITC dye	I.V.	SPF/VAF outbred rats, Balb/c mice	In vivo imaging	[35]
	Iron oxide nanoparticles	20 nm	Dextran			S.C.	Wistar normal rats	In vivo imaging	[36]
Inorganic	Silica nanoparticles	77 nm		CpG DNA, ovalbumin		S.C.	C57BL/6 mice bearing EG.7-OVA tumor	In vivo efficacy	[37]
	NaGdF4:Yb,Er@NaGdF4	18.6 ± 0.9 nm	MGb2 antibody			I.V.	BALB/c nude mice bearing SGC7901-Luc tumor	In vivo imaging	[38]
	W18O49	4.5 nm	HER-2 antibody			S.C.	Balb/c nude mice bearing MM435/Luc tumor	In vivo efficacy	[39]
Carbon	Multi-walled carbon nanotubes	40~60 nm	Polyacrylic acid, Fe_3_O_4_ nanoparticles	Gemcitabine		S.C.	Balb/c nude mice bearing BxPC-3 tumor or SW1990 tumor	In vivo efficacy	[40]

**Table 2:** Clinically approved nanoparticles.

Tracer	Detection Method	Trade Name
Iodine	X-ray	Lipiodol Ultra-Fluid
Technetium-99m	SPECT	Technecoll
Iron oxide	MR imaging	MagTrace, COMBIDEX
Perflutren	US imaging	Optison

## Data Availability

No new data were created or analyzed in this study. Data sharing is not applicable to this article.

## References

[1] Schudel A., Francis D.M., Thomas S.N. (2019). Material design for lymph node drug delivery. Nat. Rev. Mater..

[2] Chang J.E., Turley S.J. (2015). Stromal infrastructure of the lymph node and coordination of immunity. Trends Immunol..

[3] Achen M.G., Stacker S.A. (2008). Molecular control of lymphatic metastasis. Ann. N. Y. Acad. Sci..

[4] Mariani G., Moresco L., Viale G., Villa G., Bagnasco M., Canavese G., Buscombe J., Strauss H.W., Paganelli G. (2001). Radioguided sentinel lymph node biopsy in breast cancer surgery. J. Nucl. Med..

[5] Li P., Sun D. (2022). Advanced diagnostic imaging of sentinel lymph node in early stage breast cancer. J. Clin. Ultrasound.

[6] Polom K., Murawa D., Rho Y.S., Nowaczyk P., Hünerbein M., Murawa P. (2011). Current trends and emerging future of indocyanine green usage in surgery and oncology: A literature review. Cancer.

[7] Miao Y., Gu C., Zhu Y., Yu B., Shen Y., Cong H. (2018). Recent Progress in Fluorescence Imaging of the Near-Infrared II Window. ChemBioChem.

[8] Pieper C.C., Hur S., Sommer C.-M., Nadolski G., Maleux G., Kim J., Itkin M. (2019). Back to the Future: Lipiodol in Lymphography—From Diagnostics to Theranostics. Investig. Radiol..

[9] van Leeuwen F.W.B., Winter A., van Der Poel H.G., Eiber M., Suardi N., Graefen M., Wawroschek F., Maurer T. (2019). Technologies for image-guided surgery for managing lymphatic metastases in prostate cancer. Nat. Rev. Urol..

[10] Sakorafas G.H., Peros G., Cataliotti L., Vlastos G. (2006). Lymphedema following axillary lymph node dissection for breast cancer. Surg. Oncol..

[11] Porter C.J.H., Trevaskis N.L. (2020). Targeting immune cells within lymph nodes. Nat. Nanotechnol..

[12] Jeong S.H., Jang J.H., Cho H.Y., Lee Y.B. (2018). Soft- and hard-lipid nanoparticles: A novel approach to lymphatic drug delivery. Arch. Pharm. Res..

[13] Wang Z., Xia H., Chen B., Wang Y., Yin Q., Yan Y., Yang Y., Tang M., Liu J., Zhao R. (2021). pH-Amplified CRET Nanoparticles for In Vivo Imaging of Tumor Metastatic Lymph Nodes. Angew. Chem. Int. Ed..

[14] Bennett Z.T., Feng Q., Bishop J.A., Huang G., Sumer B.D., Gao J. (2020). Detection of Lymph Node Metastases by Ultra-pH-Sensitive Polymeric Nanoparticles. Theranostics.

[15] McCright J., Skeen C., Yarmovsky J., Maisel K. (2022). Nanoparticles with dense poly(ethylene glycol) coatings with near neutral charge are maximally transported across lymphatics and to the lymph nodes. Acta Biomater..

[16] Schudel A., Chapman A.P., Yau M.-K., Higginson C.J., Francis D.M., Manspeaker M.P., Avecilla A.R.C., Rohner N.A., Finn M.G., Thomas S.N. (2020). Programmable multistage drug delivery to lymph nodes. Nat. Nanotechnol..

[17] Kang T., Zhu Q., Wei D., Feng J., Yao J., Jiang T., Song Q., Wei X., Chen H., Gao X. (2017). Nanoparticles Coated with Neutrophil Membranes Can Effectively Treat Cancer Metastasis. ACS Nano.

[18] Gong H., Chao Y., Xiang J., Han X., Song G., Feng L., Liu J., Yang G., Chen Q., Liu Z. (2016). Hyaluronidase To Enhance Nanoparticle-Based Photodynamic Tumor Therapy. Nano Lett..

[19] Tsujimoto H., Morimoto Y., Takahata R., Nomura S., Yoshida K., Hiraki S., Horiguchi H., Miyazaki H., Ono S., Saito D. (2015). Theranostic Photosensitive Nanoparticles for Lymph Node Metastasis of Gastric Cancer. Ann. Surg. Oncol..

[20] Cabral H., Makino J., Matsumoto Y., Mi P., Wu H., Nomoto T., Toh K., Yamada N., Higuchi Y., Konishi S. (2015). Systemic Targeting of Lymph Node Metastasis through the Blood Vascular System by Using Size-Controlled Nanocarriers. ACS Nano.

[21] Jeanbart L., Ballester M., de Titta A., Corthésy P., Romero P., Hubbell J.A., Swartz M.A. (2014). Enhancing efficacy of anticancer vaccines by targeted delivery to tumor-draining lymph nodes. Cancer Immunol. Res..

[22] Khullar O.V., Griset A.P., Gibbs-Strauss S.L., Chirieac L.R., Zubris K.A.V., Frangioni J.V., Grinstaff M.W., Colson Y.L. (2012). Nanoparticle Migration and Delivery of Paclitaxel to Regional Lymph Nodes in a Large Animal Model. J. Am. Coll. Surg..

[23] Luo G., Yu X., Jin C., Yang F., Fu D., Long J., Xu J., Zhan C., Lu W. (2010). LyP-1-conjugated nanoparticles for targeting drug delivery to lymphatic metastatic tumors. Int. J. Pharm..

[24] Dai Y., Yu X., Wei J., Zeng F., Li Y., Yang X., Luo Q., Zhang Z. (2020). Metastatic status of sentinel lymph nodes in breast cancer determined with photoacoustic microscopy via dual-targeting nanoparticles. Light Sci. Appl..

[25] Mei L., Rao J., Liu Y., Li M., Zhang Z., He Q. (2018). Effective treatment of the primary tumor and lymph node metastasis by polymeric micelles with variable particle sizes. J. Control. Release.

[26] Kim K.S., Lee S., Na K., Bae Y.H. (2022). Ovalbumin and Poly(i:c) Encapsulated Dendritic Cell-Targeted Nanoparticles for Immune Activation in the Small Intestinal Lymphatic System. Adv. Healthc. Mater..

[27] Liu H., Moynihan K.D., Zheng Y., Szeto G.L., Li A.V., Huang B., Van Egeren D.S., Park C., Irvine D.J. (2014). Structure-based programming of lymph-node targeting in molecular vaccines. Nature.

[28] Cai X., Liu X., Liao L.-D., Bandla A., Ling J.M., Liu Y.-H., Thakor N., Bazan G.C., Liu B. (2016). Encapsulated Conjugated Oligomer Nanoparticles for Real-Time Photoacoustic Sentinel Lymph Node Imaging and Targeted Photothermal Therapy. Small.

[29] Muhanna N., MacDonald T.D., Chan H., Jin C.S., Burgess L., Cui L., Chen J., Irish J.C., Zheng G. (2015). Multimodal Nanoparticle for Primary Tumor Delineation and Lymphatic Metastasis Mapping in a Head-and-Neck Cancer Rabbit Model. Adv. Healthc. Mater..

[30] Kato S., Mori S., Kodama T. (2015). A Novel Treatment Method for Lymph Node Metastasis Using a Lymphatic Drug Delivery System with Nano/Microbubbles and Ultrasound. J. Cancer.

[31] Tseng Y.-C., Xu Z., Guley K., Yuan H., Huang L. (2014). Lipid–calcium phosphate nanoparticles for delivery to the lymphatic system and SPECT/CT imaging of lymph node metastases. Biomaterials.

[32] Shi H., Yan R., Wu L., Sun Y., Liu S., Zhou Z., He J., Ye D. (2018). Tumor-targeting CuS nanoparticles for multimodal imaging and guided photothermal therapy of lymph node metastasis. Acta Biomater..

[33] Uthaman S., Kim H.S., Revuri V., Min J.-J., Lee Y.-K., Huh K.M., Park I.-K. (2018). Green synthesis of bioactive polysaccharide-capped gold nanoparticles for lymph node CT imaging. Carbohydr. Polym..

[34] Kang S., Ahn S., Lee J., Kim J.Y., Choi M., Gujrati V., Kim H., Kim J., Shin E.C., Jon S. (2017). Effects of gold nanoparticle-based vaccine size on lymph node delivery and cytotoxic T-lymphocyte responses. J. Control. Release.

[35] Vu-Quang H., Yoo M.-K., Jeong H.-J., Lee H.-J., Muthiah M., Rhee J.H., Lee J.-H., Cho C.-S., Jeong Y.Y., Park I.-K. (2011). Targeted delivery of mannan-coated superparamagnetic iron oxide nanoparticles to antigen-presenting cells for magnetic resonance-based diagnosis of metastatic lymph nodes in vivo. Acta Biomater..

[36] Oghabian M.A., Gharehaghaji N., Amirmohseni S., Khoei S., Guiti M. (2010). Detection sensitivity of lymph nodes of various sizes using USPIO nanoparticles in magnetic resonance imaging. Nanomed. Nanotechnol. Biol. Med..

[37] An M., Li M., Xi J., Liu H. (2017). Silica Nanoparticle as a Lymph Node Targeting Platform for Vaccine Delivery. ACS Appl. Mater. Interfaces.

[38] Qiao R., Liu C., Liu M., Hu H., Liu C., Hou Y., Wu K., Lin Y., Liang J., Gao M. (2015). Ultrasensitive in Vivo Detection of Primary Gastric Tumor and Lymphatic Metastasis Using Upconversion Nanoparticles. ACS Nano.

[39] Huo D., He J., Li H., Huang A.J., Zhao H.Y., Ding Y., Zhou Z.Y., Hu Y. (2014). X-ray CT guided fault-free photothermal ablation of metastatic lymph nodes with ultrafine HER-2 targeting W18O49 nanoparticles. Biomaterials.

[40] Yang F., Jin C., Yang D., Jiang Y., Li J., Di Y., Hu J., Wang C., Ni Q., Fu D. (2011). Magnetic functionalised carbon nanotubes as drug vehicles for cancer lymph node metastasis treatment. Eur. J. Cancer.

[41] Zhou K., Wang Y., Huang X., Luby-Phelps K., Sumer B.D., Gao J. (2011). Tunable, Ultrasensitive pH-Responsive Nanoparticles Targeting Specific Endocytic Organelles in Living Cells. Angew. Chem. Int. Ed..

[42] Kim M.-H., Nguyen D.-T., Kim D.-D. (2022). Recent studies on modulating hyaluronic acid-based hydrogels for controlled drug delivery. J. Pharm. Investig..

[43] Tan T., Hu H., Wang H., Li J., Wang Z., Wang J., Wang S., Zhang Z., Li Y. (2019). Bioinspired lipoproteins-mediated photothermia remodels tumor stroma to improve cancer cell accessibility of second nanoparticles. Nat. Commun..

[44] Tsopelas C., Sutton R. (2002). Why Certain Dyes Are Useful for Localizing the Sentinel Lymph Node. J. Nucl. Med..

[45] Markuszewski M., Buszewska-Forajta M., Artymowicz M., Połom W., Roslan M., Markuszewski M. (2022). Binding indocyanine green to human serum albumin potentially enhances the detection of sentinel lymph nodes. An initial step for facilitating the detection of first-station nodes in penile and other urological cancers. Arch. Med. Sci..

[46] Pavlista D., Eliska O. (2012). Analysis of direct oil contrast lymphography of upper limb lymphatics traversing the axilla—A lesson from the past—Contribution to the concept of axillary reverse mapping. Eur. J. Surg. Oncol..

[47] Karakatsanis A., Daskalakis K., Stålberg P., Olofsson H., Andersson Y., Eriksson S., Bergkvist L., Wärnberg F. (2017). Superparamagnetic iron oxide nanoparticles as the sole method for sentinel node biopsy detection in patients with breast cancer. Br. J. Surg..

[48] Winter A., Woenkhaus J., Wawroschek F. (2014). A novel method for intraoperative sentinel lymph node detection in prostate cancer patients using superparamagnetic iron oxide nanoparticles and a handheld magnetometer: The initial clinical experience. Ann. Surg. Oncol..

[49] Winter A., Kowald T., Paulo T.S., Goos P., Engels S., Gerullis H., Schiffmann J., Chavan A., Wawroschek F. (2018). Magnetic resonance sentinel lymph node imaging and magnetometer-guided intraoperative detection in prostate cancer using superparamagnetic iron oxide nanoparticles. Int. J. Nanomed..

[50] Gu J., Wang J., Nie X., Wang W., Shang J. (2015). Potential role for carbon nanoparticles identification and preservation in situ of parathyroid glands during total thyroidectomy and central compartment node dissection. Int. J. Clin. Exp. Med..

[51] Wu G., Cai L., Hu J., Zhao R., Ge J., Zhao Y., Shi J., Wang Z. (2015). Role of carbon nanoparticles in patients with thyroid carcinoma undergoing total thyroidectomy plus bilateral central neck dissection. Zhonghua Yi Xue Za Zhi.

[52] Xue S., Ren P., Wang P., Chen G. (2018). Short and Long-Term Potential Role of Carbon Nanoparticles in Total Thyroidectomy with Central Lymph Node Dissection. Sci. Rep..

[53] Tenchov R., Bird R., Curtze A.E., Zhou Q. (2021). Lipid Nanoparticles—From Liposomes to mRNA Vaccine Delivery, a Landscape of Research Diversity and Advancement. ACS Nano.

[54] Angelova A., Angelov B. (2017). Dual and multi-drug delivery nanoparticles towards neuronal survival and synaptic repair. Neural Regen. Res..

